# Polycystins, mechanotransduction and cancer development

**DOI:** 10.1111/jcmm.17298

**Published:** 2022-04-02

**Authors:** Kostas A. Papavassiliou, Antonios N. Gargalionis, Athanasios G. Papavassiliou

**Affiliations:** ^1^ Department of Biological Chemistry Medical School National and Kapodistrian University of Athens Athens Greece

**Keywords:** cancer, mechanotransduction, polycystins, solid tumour

Solid tumours represent the most frequent type of tumours worldwide, and they are responsible for high morbidity and mortality rates.^1^ There is an ongoing and compelling need for novel prognostic, diagnostic and predictive markers to tailor personalized treatments, establish concrete molecular classifications of the tumours and discover new therapeutic targets. Accumulating evidence indicates that mechanical stimuli and physical forces modulate cancer cell behaviour, mainly through the process of mechanotransduction.

An ever‐increasing volume of data shed light on the effect of abnormal mechanically‐induced stimulation of the extracellular matrix (ECM) and corresponding distorted mechanotransduction of cancer cells and neighbouring stromal cells in tumorigenesis.^2^, ^3^ Among others, perverted tissue mechanics has been linked to tumour invasion and metastasis.^2^, ^4^ The influence of tumour mechanobiology on cancer features opens potential new therapeutic horizons; thus, marking a new field named mechanopharmacology, which intents to exploit tumour–microenvironment physical associations.^2^, ^5^

Besides genetic mutations, other factors including chronic inflammation, tissue injury and accumulated mechanical stress reprogramme and activate differentiating regulatory circuits that were normally silent in adults.^3^ Such events lead to irregular cell‐to‐cell communication and cell‐to‐ECM interactions, loss of epithelial‐to‐mesenchymal balance, atypical ECM turnover and anomalous mechanotransduction. Aberrant mechanotransduction leads to altered intracellular signalling as a response to augmented ECM stiffness, which affects gene transcription in favour of cancer initiation and progression.^3^ The above mechanisms are mediated through core proteins of the adhesive complex, predominantly via integrin/focal adhesion kinase (FAK)/Src signalling, which foster cytoskeletal remodelling, development of increased intracellular tension, ultimately generating positive feedback loops and a vicious circle between the ECM and the cytoskeleton.^3^ They are also mediated through the mechano‐induced transcriptional co‐activators of the Hippo signalling cascade, Yes‐associated protein (YAP)/transcriptional co‐activator with PDZ‐binding motif (TAZ).^6^

The term polycystins refers to a relatively new family of proteins that comprises eight protein species. Two representative members of the family are polycystin‐1 (PC1) and polycystin‐2 (PC2), which are encoded by the polycystic kidney disease 1 (PKD1) and polycystic kidney disease 2 (PKD2) genes respectively.^7^ Polycystins constitute a group of proteins that have come to the fore as major mechanosensitive molecules in epithelial cells. PC1 and PC2 were first identified as causative agents of autosomal dominant polycystic kidney disease (ADPKD), the most frequent inherited kidney disorder worldwide.^8^

Polycystin‐1 is a transmembrane protein bearing a long, flexible, mechanosensitive N‐terminus and an intracellular C‐terminus that gives rise to transcriptionally active peptide fragments. It operates as a mechanosensor that perceives extracellular mechanical cues and regulates vital cellular functions such as proliferation, differentiation and apoptosis.^9^, ^10^ PC2 is an indiscriminate cation channel permeable to Ca^2+^ ions, which belongs to the family of transient receptor potential (TRP) channels.^10^, ^11^ PC1 is expressed in a wide variety of tissues in the human body. The expression is usually restricted to epithelial cells of tissues, such as in the urothelium of the bladder epithelium, in the liver, breast and pancreas.^12^ Elevated levels of PC1 expression are found in the cerebral cortex, astrocytes in particular.^13^, ^14^ PC2 is diffusely expressed in a broad spectrum of tissues, especially in the nerve ganglia of the neural tube, liver and myocardium, whereas the expression at the 16th week of organogenesis is strongest in the anterior roots of the spinal cord.^15^, ^16^ PC1 and PC2 interact physically in vivo via their C‐terminus generating heterodimeric complexes at the cell membrane and at epithelial formations known as primary cilia. This ‘aggregate’ creates non‐selective cation channels pervious to Ca^2+^ ions.^17^, ^18^

Among the biological properties that characterize cancer cells is the continuous transmission of signals that promote proliferation, resistance to apoptosis and activation of invasive mechanisms and metastasis.^19^ Participation of polycystins in pivotal cellular functions such as proliferation, apoptosis, mediation of intercellular interactions, communication with the ECM and cell orientation, suggest the potential contribution of this family of proteins to tumorigenic processes, especially invasion and metastasis.^11^, ^16^

Polycystins are involved in the control of cellular homeostasis, particularly during embryonic morphogenesis and regeneration of differentiated tissues by regulating the cell‐cycle, intercellular interactions and interactions with the ECM. PC1 is mainly located at multiple focal adhesion structures, the primary cell structures that mediate communication of cells with the ECM.^10^ Accordingly, it has been detected in complexes with alpha‐actinin, vinculin, tensin, talin and signalling proteins such as FAK, Src, p130Cas and paxillin in epithelial and smooth muscle cells, which are the molecules that steer mechanotransduction.^10^, ^20^ Thus, a model has been proposed that implicates polycystins in cancer progression, where PC2 regulates Ca^2+^ influx and PC1 acts as a membrane modulator of cell‐to‐cell and cell‐to‐ECM interactions with a putative capacity of modifying oncogenic transcription programmes.^3^, ^11^

Increased expression of polycystins in the cystic epithelium as a reflection of the dedifferentiation of the tissue and the perception of cysteogenesis as a multistep process that requires the progressive accumulation of genetic alterations, have justifiably attributed to ADPKD the term ‘neoplasia in disguise’.^21^ Studies reveal the primary cilia to participate in carcinogenesis, a fact that generated a theory which describes the mechano‐induced signal transduction networks mediated by primary cilia as a component of tumorigenesis.^22^ Both in medulloblastoma, the most common brain tumour in children, and in the basal carcinoma of the skin, absence of primary cilia proved that either leads to inhibition of, or contributes to tumour growth depending on whether the origin of tumorigenesis is due to inarticulate activation of protein Smoothened (Smo) of the Hedgehog signalling pathway, or activation of the transcription factor Gli2 of the same pathway respectively.^23^, ^24^ Moreover, the study of human glioblastoma cell lines revealed damage to the structure and function of primary cilia.^25^

Few studies have been published discussing the relationship of polycystins with carcinogenesis. Overexpression of PC1 in lung cancer, colon cancer and hepatocellular carcinoma cell lines led to promotion of ECM and intercellular interactions, thus inhibiting invasion and migration of tumour cells via the Wnt pathway, attributing to PC1 a potential tumour suppressing role.^26^ Reduced expression of PC2 employing silencing RNAs (siRNAs) resulted in significant suppression of intercellular adhesion in melanoma cells from B16 mice.^27^ These two studies show that PC1 and PC2 affect cell adhesion processes and communication with the ECM in cancer cells. PC1 overexpression in the aforementioned cell lines resulted in a substantial increase in apoptosis and also cell‐cycle arrest in G0/G1 phase, suggesting its involvement in cell‐cycle regulation in tumour cells.^16^, ^26^, ^28^

Strong evidence for the validity of this model comes from another study where PC1 and PC2 were demonstrated to be engaged in the development of aggressive phenotypes in colorectal cancer (CRC).^17^, ^29^ Specifically, the overexpression of PC1 promotes epithelial‐to‐mesenchymal transition (EMT), whilst PC2 overexpression triggers activation of the mechanistic target of rapamycin (mTOR) pathway. Functional hampering of the extracellular domain of PC1 reduces cell proliferation and suppresses EMT, but also promotes tumour necrosis in HT29 xenografts. Augmented expression of PC1 and PC2 was associated with aggressive phenotypes, while PC1 emerged as an independent predictor of survival without relapse.^10^, ^17^, ^29^ PC1 was also recently found to interact with the mechano‐induced transcriptional co‐activator TAZ via its C‐terminal tail during osteoblastic differentiation.^30^ This is important because the YAP/TAZ transcription co‐factors integrate physical‐associated cancer mechanisms.^6^ Furthermore, it has been reported that PC1 modulation impacts proliferation and migration of MG‐63 osteosarcoma cells in a bone‐derived tumour that develops with increased stiffness and is subjected to mechanical stretching through potentiation of mechanosensitive focal adhesion molecules.^2^, ^31^ Therefore, polycystins come into sight as key mechanosensors in cancer biomechanics that can integrate multiple oncogenic signalling networks. Accordingly, polycystins emerge as novel protein species that may serve as potential therapeutic targets and/or biomarkers emanated from the flourishing field of tumour mechanobiology.^2^

Mechanotransduction and the overall investigation of the contribution of the mechanical forces exerted on normal cells and also on tumour cells during the development and invasion of solid tumours is a rapidly evolving research area over the past few years. Nevertheless, the exact molecular mechanisms by which mechanotransduction is involved in tumorigenesis remain a challenge. Polycystins are decisive components of mechanotransduction that regulate a wide gamut of cellular functions during embryogenesis and adult life.

High heterogeneity of solid tumours, even among the same tumour cells, is a major obstacle to the development of effective treatment strategies. Even newly developed remedies demonstrate acquired resistance after a certain period of time; hence, elucidation of the molecular mechanisms underpinning the polycystins/TRP channels‐funneled mechanotransduction process is essential for the discovery of novel diagnostic, prognostic and therapeutic tools,^11^, ^,32^, ^,33^ especially predictive biomarkers.

## CONFLICT OF INTEREST

The authors confirm that there are no conflicts of interest.

## AUTHOR CONTRIBUTIONS

**Kostas A. Papavassiliou:** Data curation (lead); Writing – original draft (lead). **Antonios N. Gargalionis:** Data curation (lead); Writing – original draft (lead). **Athanasios G. Papavassiliou:** Conceptualization (lead); Supervision (lead); Validation (lead); Writing – review & editing (lead).
